# Supplementary figures and images for: Antibiotic Use for Common Infections in Pediatric Emergency Departments: A Narrative Review
Source: Antibiotics (Basel). 2023 Jun 22;12(7):1092. doi: 10.3390/antibiotics12071092 (PMC10376281; doi:10.3390/antibiotics12071092)

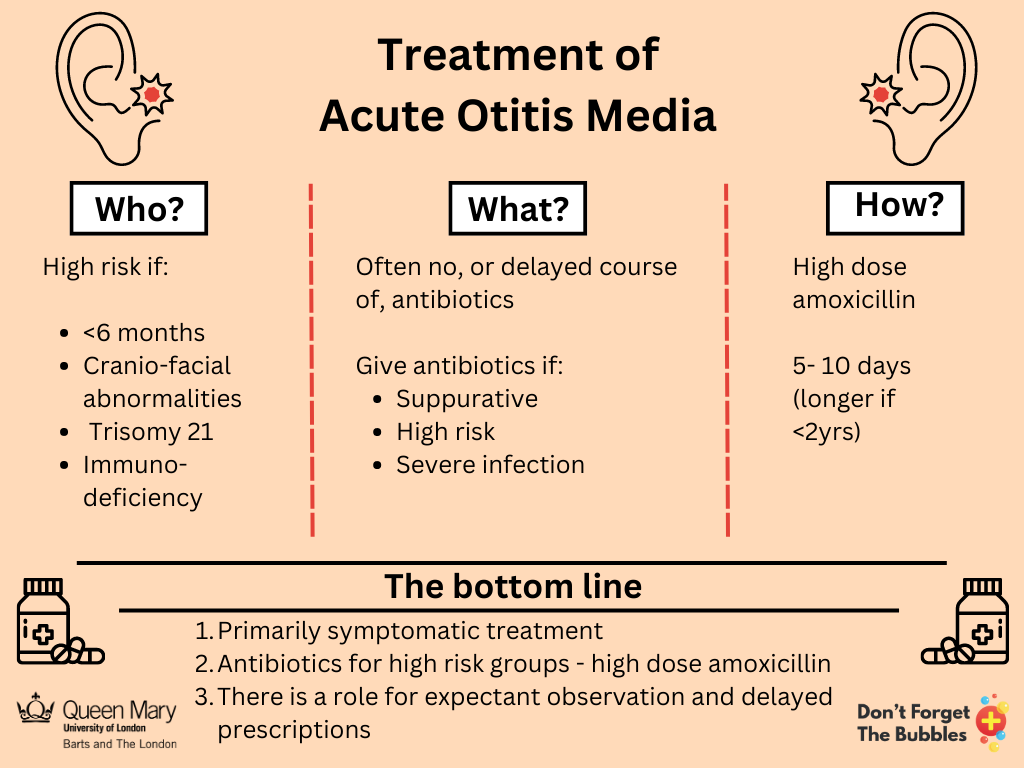

Supplement: Supplementary file 1 [file antibiotics-12-01092-s001.zip › Acute Otitis Media Figure S1.png]

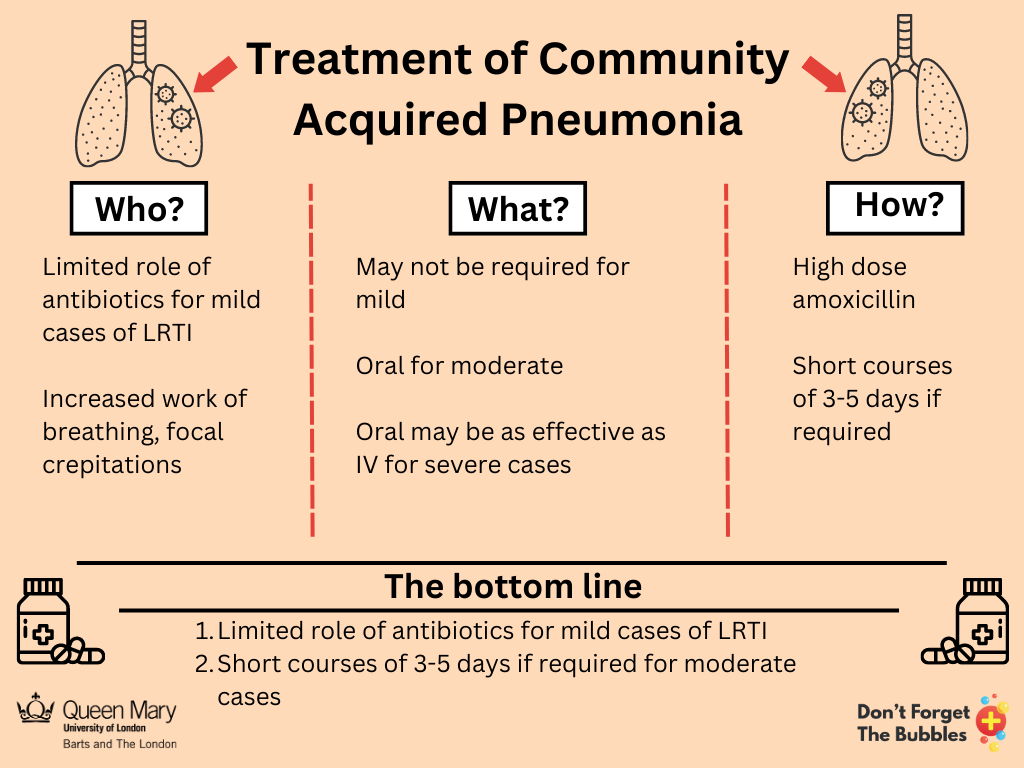

Supplement: Supplementary file 1 [file antibiotics-12-01092-s001.zip › Community-acquired Pneumonia Figure S3.png]

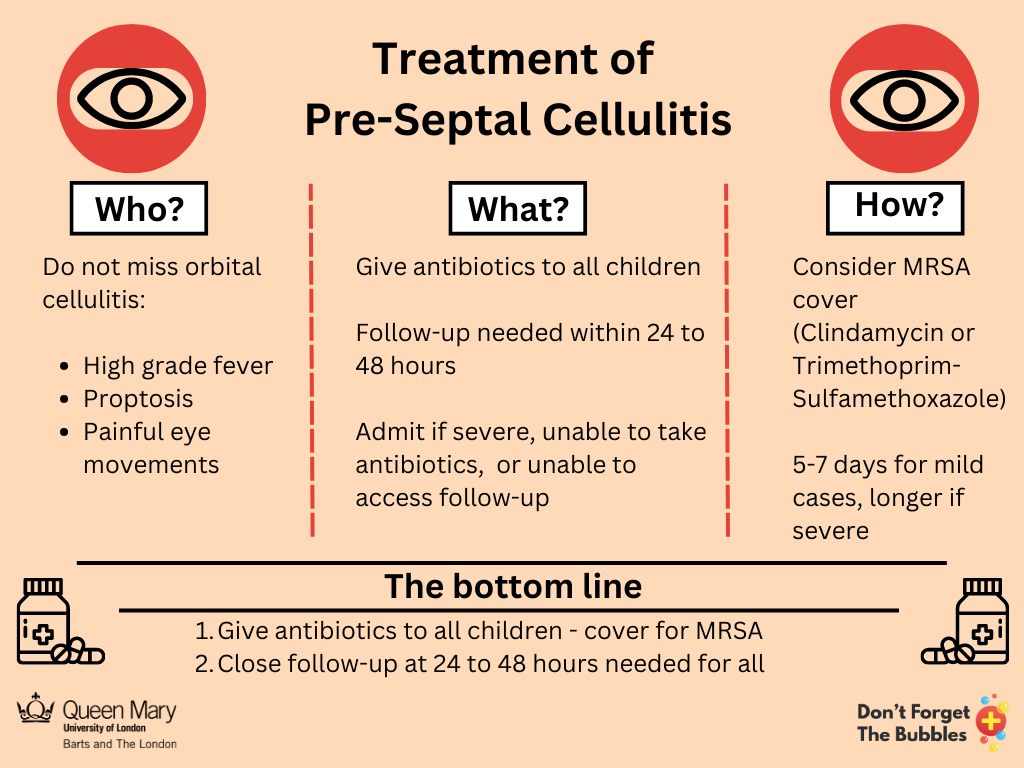

Supplement: Supplementary file 1 [file antibiotics-12-01092-s001.zip › Preseptal cellulitis Figure S4.png]

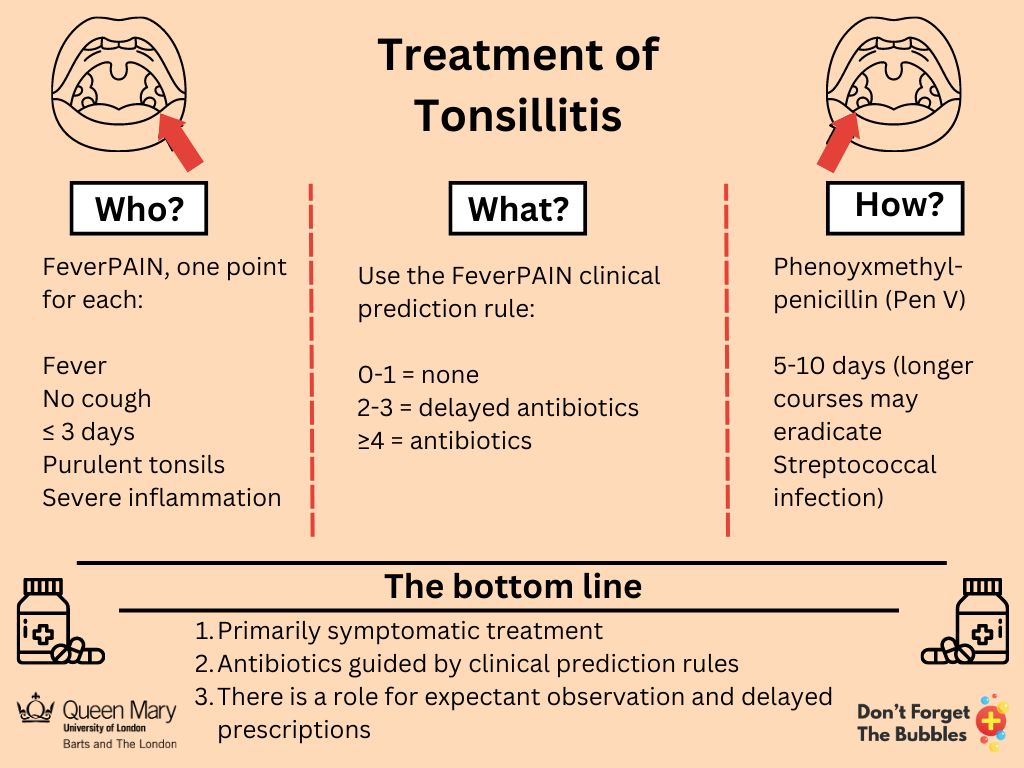

Supplement: Supplementary file 1 [file antibiotics-12-01092-s001.zip › Tonsillitis Figure S2.png]

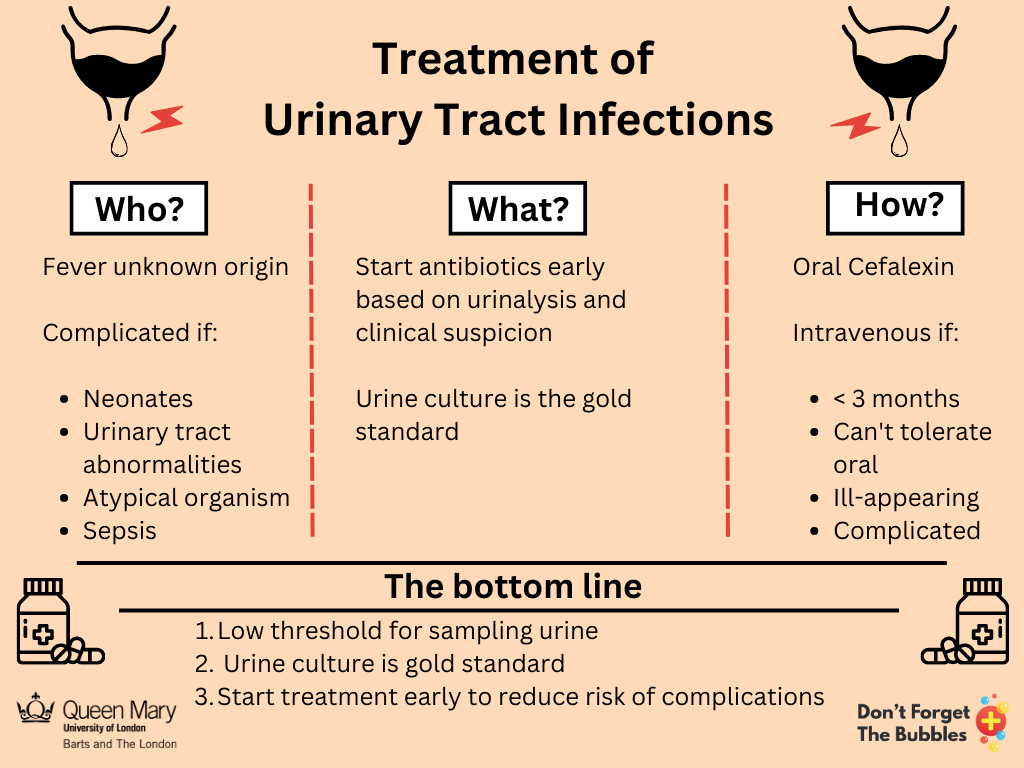

Supplement: Supplementary file 1 [file antibiotics-12-01092-s001.zip › Urinary tract infections Figure S5.png]
